# [Retracted] MicroRNA‑526a targets p21‑activated kinase 7 to inhibit tumorigenesis in hepatocellular carcinoma

**DOI:** 10.3892/mmr.2023.13007

**Published:** 2023-05-09

**Authors:** Lei Zhan, Yaozhen Pan, Ling Chen, Zili Chen, Hong Zhang, Chengyi Sun

Mol Med Rep 16: 837–844, 2017; DOI: 10.3892/mmr.2017.6658

Following the publication of this paper, it was drawn to the Editors’ attention by a concerned reader that certain of the data shown for the Transwell cell migration and invasion assays in Fig. 2C and D were strikingly similar to data appearing in different form in other articles by different authors. Owing to the fact that the contentious data in the above article had already been published elsewhere prior to its submission to *Molecular Medicine Reports*, the Editor has decided that this paper should be retracted from the Journal. The authors were asked for an explanation to account for these concerns, but the Editorial Office did not receive a reply. The Editor apologizes to the readership for any inconvenience caused.

